# Obesity, Inflammation, and Immune System in Osteoarthritis

**DOI:** 10.3389/fimmu.2022.907750

**Published:** 2022-07-04

**Authors:** Udhaya Nedunchezhiyan, Ibin Varughese, Antonia RuJia Sun, Xiaoxin Wu, Ross Crawford, Indira Prasadam

**Affiliations:** ^1^ Centre for Biomedical Technologies, Faculty of Engineering, Queensland University of Technology, Brisbane, QLD, Australia; ^2^ Department of Orthopedic Surgery, The Second Xiangya Hospital, Central South University, Changsha, China; ^3^ Orthopedic Department, The Prince Charles Hospital, Brisbane, QLD, Australia

**Keywords:** osteoarthritis, obesity, innate immunity, adaptive immunity, synovium & osteoarthritis, biomechanics, T cells

## Abstract

Obesity remains the most important risk factor for the incidence and progression of osteoarthritis (OA). The leading cause of OA was believed to be overloading the joints due to excess weight which in turn leads to the destruction of articular cartilage. However, recent studies have proved otherwise, various other factors like adipose deposition, insulin resistance, and especially the improper coordination of innate and adaptive immune responses may lead to the initiation and progression of obesity-associated OA. It is becoming increasingly evident that multiple inflammatory cells are recruited into the synovial joint that serves an important role in pathological changes in the synovial joint. Polarization of macrophages and macrophage-produced mediators are extensively studied and linked to the inflammatory and destructive responses in the OA synovium and cartilage. However, the role of other major innate immune cells such as neutrophils, eosinophils, and dendritic cells in the pathogenesis of OA has not been fully evaluated. Although cells of the adaptive immune system contribute to the pathogenesis of obesity-induced OA is still under exploration, a quantity of literature indicates OA synovium has an enriched population of T cells and B cells compared with healthy control. The interplay between a variety of immune cells and other cells that reside in the articular joints may constitute a vicious cycle, leading to pathological changes of the articular joint in obese individuals. This review addresses obesity and the role of all the immune cells that are involved in OA and summarised animal studies and human trials and knowledge gaps between the studies have been highlighted. The review also touches base on the interventions currently in clinical trials, different stages of the testing, and their shortcomings are also discussed to understand the future direction which could help in understanding the multifactorial aspects of OA where inflammation has a significant function.

## 1 Introduction

Obesity and OA are two major health problems prevalent in our society today. According to the Australian Institute of Health and Welfare, 2 in 3 (67%) of the adult population were overweight or obese in 2018 (1). It is reported that 1 in 3 (6.9 million) people suffer from musculoskeletal conditions, including arthritis which constitutes the top disease burden (2). Obesity increases the risk of OA in both weight-bearing (knee) and non-weight-bearing (hand) joints, and obesity doubles the lifetime risk of symptomatic OA compared to individuals with a BMI below 25 (3). The dynamic environment of joints is constantly subjected to mild damage through motions, and in some joints, weight-bearing (knee and hip) leads to compression, resulting in a state of persistent wound healing and repair processes. As a result, the articular cartilage and neighboring bone must continually rebuild where synthesis and degradation are a constant process (4). These activities necessitate the activation of anabolic and catabolic enzymes in bones and cartilage. Traditionally, the pathogenesis of OA was considered non-inflammatory in origin, with mechanical stress leading to cartilage destruction (5). However, recent studies suggest that OA has an inflammatory component with inflammatory cell infiltration of the synovial membrane (6–8). Recent research highlights that obesity and increased periprosthetic infections are strongly linked according to Australian Orthopaedic Association registry data, and the risk increases for the morbidly obese (9). This data highlights that the obese environment is highly complex at both the systematic and local levels. Adipose tissue (AT) plays a crucial role in the regulation of metabolic activities storing excess energy as triglycerides and converting them into fatty acids and glycerol, in required places. They also perform an additional role in secreting adipokines. Adipokines are a key player that regulates the homeostasis in inflammation, immunity, reproduction, angiogenesis, fibrinolysis, regulating appetite, coagulation, and insulin sensitivity (10). It is becoming evident with piles of novel studies that correlate obesity-induced adipokine production which leads to the onset of OA (11, 12). A large genome-wide associated study was run to identify the susceptibility risk loci. The study found that over 140 genes were associated with OA and majority of the variants were localized in the non-coding region which made it inconspicuous. Hence, it is essential to establish the functional link between genomic and disease-relevant alteration on multimolecular levels (13–15).

### 1.1 Comparative View on the Healthy Synovial Microenvironment and OA Synovium Microenvironment

Synovium is a soft tissue found in the diarthrodial joints, tendon sheaths and bursae. They have consecutive layers of cells, where inner layer is called the intima and the outer layer is called the subintima. The intimal layer houses the macrophages and fibroblasts while the subintima has blood and lymphatic vessels, fibroblast and infiltration of cells in a collagenous extracellular matrix (16). The intimal layer is 20- 40mm thick in cross-section and the subintima can be up to 5mm in thickness (16, 17). The synovium is responsible for maintaining the functional activity of articular cartilage and the well-being of chondrocytes by producing lubricin and hyaluronic acid (i.e. synovial fluid) (16). Numerous factors contribute to synovial joint homeostasis, including regular expression of protective lubricin, fibroblast-like synoviocytes (FLS) secretion of matrix metalloproteinase (MMPs), immune centralized role is played by resident macrophages and FLS, regulated entry and exit of leukocytes involved in immune surveillance, and local regulation by cytokines and growth factors (4).

Synovitis is increasingly recognized as a prevalent symptom of OA, both in early and late stages, and as such, it provides a potential target for treatment, both for symptom relief and structural alteration. OA synovial tissue (ST) has a lower overall inflammatory profile than Rheumatoid arthritis (RA), although it is greater than healthy controls. From the synovial membranes of OA patients, a range of immune cells from both the innate and adaptive immune families have been discovered. Lindblad et al. found that inflammation in the synovium adjacent to cartilage elicited a stronger inflammatory response, with T cells surrounded by B cells and plasma cells (18). Revell et al. observed lymphoid follicle growth in OA synovial membrane throughout the same period, emphasizing the relevance of B lymphocytes and granulocytes in the pathogenesis of OA (19)

## 2 Role of Innate Immunity in Obesity and OA

The innate immune system is a primitive mechanism that humans inherit from invertebrates that use germline-encoded proteins to recognize pathogens. Upon exposure to these pathogens, the innate immune cells either kill the pathogens directly or recruit the adaptive immune system through a series of events. Cells of the innate immune system consist of macrophages, dendritic cells (DCs), and natural killer cells (NK) which recognize Pathogen-Associated Molecular Patterns (PAMPs) or Damage-Associated Molecular Patterns (DAMPs) that originate from highly conserved parts of microbes and use a diverse set of patterns recognition receptors (PRR) molecules like lipopolysaccharide (LPS) (20). One of the potential mechanisms of LPS leakage into the systemic circulation and sensitization of the immune system is through dysbiosis gut-associated with obesity. The leakage has been implicated in the development of low-grade inflammation both systematically through the release of LPS and locally in the small intestine and ST (21, 22).

**Figure 1:**
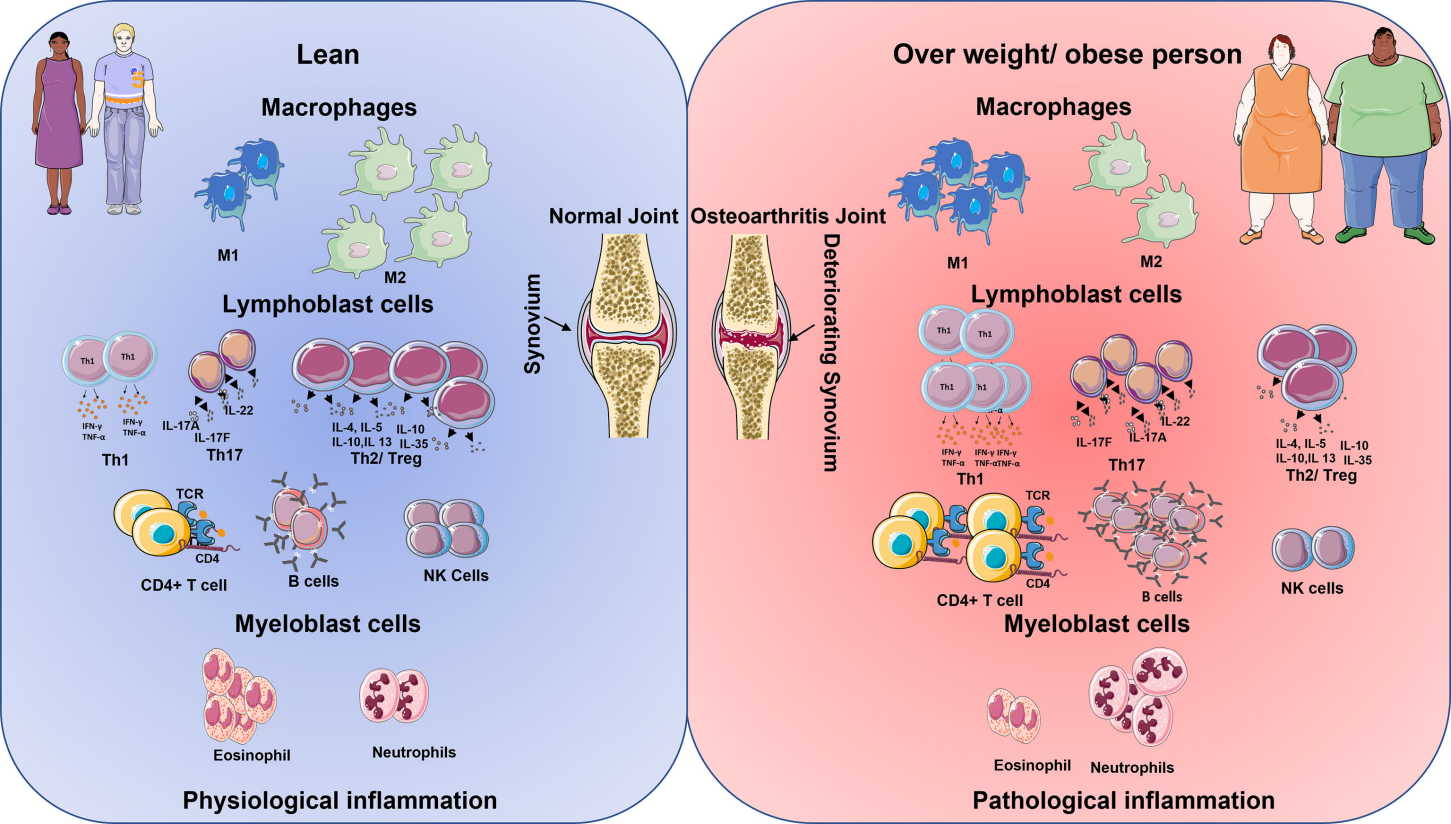
Schematic diagram shows the immune cell being up and downregulated in lean and overweight/obese individuals. The cells on the left represent the lean physiological aspects of the immune system and on the right, the obese immune system (24). In lean population during inflamed condition the macrophages M1 cells are lower in comparison to M2 cells. However, the reverse is seen in the obese inflammation. Lymphoblastic cells like Th1, Th17, T helper, and B cells are lower in comparison with Treg cells and NK cells in lean inflammation population. However, in obese population, there is an increase in the production of Th1, Th17, T helper cells, B cells but reduction in the number of T reg cells and NK becomes low. Granulocytes like the eosinophiles are higher in comparison with neutrophiles in lean inflammation condition, however, the reverse is observed with obese population in their inflammatory condition.

Classically, the innate immune system is activated by host responses to PAMPs generated by interactions with invariable pattern recognition receptors (PRRs) on synovial joint immune cells such as neutrophils, macrophages, monocytes, and DC. PRRs are a group of cell surface, endosomal, and cytosolic receptors that include Toll-like and NOD-like receptors (23). When PRRs are activated within tissues such as the joint, they initiate rapid-onset inflammatory responses, which are then followed by the commencement of adaptive immune responses and, lastly, healing responses in the case of tissue damage. The **Figure 1** shows the innate immune cells and their upregulation/downregulation in physiological inflammation and pathological inflammation.

### 2.1 Monocytes and Macrophages in Obesity and OA

In AT, macrophages are the critical mediators of inflammation and the most abundant infiltrated cells. There is four times increase in macrophage density in the obese mice model compared to lean mice models (25). In obese mouse models, macrophages are localized in the crown-like structure around the larger adipocytes (Tab.1) (26). Macrophages are heterogeneous as characterized by cytokine secretions and surface marker expression functions. They have broadly divided into M1 or classically activated macrophages and M2 or activated macrophages (27). M1, which is considered as pro-inflammatory, is induced by pro-inflammatory mediators like LPS and interferon-gamma (IFN-γ) and thereby secretes cytokines like interleukins (IL-6 and IL-1β), inducible nitric oxide synthase (iNOS), and tumor necrosis factor-alpha (TNF-α); whereas M2 is induced by IL-4 and IL-13 and secretes anti-inflammatory IL-10, IL-1 decoy receptor, and arginase which further blocks the IL-1β and iNOS activity. White AT (WAT) in obese tissue has been observed to increase the number of macrophages and mast cells compared to lean tissue leading to increased gene expression of TNF- α and a remarkable increase in IL-6 and iNOS levels (25, 28).

The cytokine expression of macrophages differs in its polarisation. Even though macrophage polarisation is not binary, it is expressed in a continuum. M1 macrophages have been shown to express IFN-γ and LPS driven macrophage phenotypes, whereas M2 refers to macrophage phenotypes triggered by IL-4 or IL-13. Furthermore, this representation reveals the mixed signals that lead to cytotoxic function of M1, anti-inflammatory, tissue remodelling, and repair from M2 macrophage polarisation (29). Regulation of macrophage polarization and functions are tightly controlled through the activation of several interconnected pathways. Among the few factors, STAT1 and STAT3/STAT6 transcription factors are crucial in the balance of macrophage activation. STAT1 activation promotes M1 polarisation, and in contrast, STAT3/6 activation by IL-4, IL-13, and IL-10 leads to increased M2 polarisation (30). In addition, IL-4 has been shown to induce c-Myc that activates the interferon regulatory factors4 (IRF4) axis, resulting in M2 promotion by inhibiting IRF5 mediated M1 polarization. IRF4 plays a role in developing a subset of myeloid cDC2 specializing in Th2 responses in mice models (31).

The characteristic differentiating surface markers of M1 differing from M2 is CD11c expression in inflammation and insulin resistance in human obesity (32). Lumeng et al. demonstrated that lean mice have predominant M2 phenotype and obese mice expressed M1 phenotype (26). However, in human obesity, multiple *in vivo* studies revealed that AT macrophages (ATM) adopt mixed M1/M2 phenotypes (30, 33). In obesity, ATM adopts a prominent metabolically activated state with increased lysosomal activity (34). This state of metabolic activation (MMe) is induced by diverse stimuli like free fatty acids, high insulin, high glucose levels (35). A “phenotypic switch” occurs in diet-induced obese mice where a shift in polarization towards M1 activation takes place over days to weeks from the M2 macrophages, which are dominant in AT of lean mice (26). ATM in an MMe are a significant source of inflammatory cytokines, and their production can be modulated by NADPH oxidase 2 activity during the progression of obesity (34). In obese condition, due to adipocyte hypertrophy, the secretion of chemo-attractants like MCP-1/CCL2 lead to macrophage recruitment and production of TNF-α, IL-6, and IL-1β, which acts as pro-inflammatory signals (35). Morris et al. demonstrated that ATM in obese models had increased expression of MHC II and T cell co-stimulatory molecules, thus processing antigens and inducing antigen specific CD4^+^ T cell population (36). ATM serves as a crucial link between innate and adaptive immunity in obesity. However, an M2 phenotypic switch can be induced through activation of PPAR-γ, thereby protecting against M1 activation and insulin resistance (37). This can potentially promote adipocyte lipid storage and prevent lipotoxicity and adipocyte death (38). De Jong et al. (39) has shown that BMI-related features of immune cell profile in subcutaneous AT (SCAT) and visceral AT (VAT) could not be detected in the infrapatellar fat pad of OA patients. Animal and clinical studies have primarily revealed that obesity does not increase the number of crown-like structures in the infrapatellar fat pad (39). Macrophages are the most widely distributed cell types in the OA synovium, mostly present along with the lining layer (40). Wood et al. reclassified OA into classical OA (cOA), which is predominantly associated with cartilage remodelling features, and inflammatory OA (iOA) subset characterized by a proliferation signature (41). Insulin-like growth factor-binding protein 5 (IGFBP-5) is overexpressed in cOA, which is associated with the negative regulation of inflammatory mediators. High-temperature requirement 1 and EGF-containing fibulin-like extracellular matrix protein 1 overexpression is observed in cOA, which modulate the synovial fibroblasts to produce cartilage catabolic MMPs and negatively regulate chondrogenesis, respectively. Macrophages in iOA were most closely aligned to macrophages associated with inflammatory arthritis, which is strongly linked to cell cycle processes. iOA macrophages have increased expression of MK167, which demonstrates the increased proliferation and chymotrypsin-like protease that reflects a pro-inflammatory environment, thus developing a positive feedback loop (41). Various DAMPs have been identified, leading to the activation of macrophages, and inducing inflammation (20).

Through etarfolatide imaging, activated macrophages are present in patients with knee OA rather than resting macrophages (42). TGF-β1 levels (2) expressed by ST macrophages in synovial fluid are found to be a strong predictor for knee OA progression (43). Recently, Wood et al. (41) described the heterogeneity of synovial macrophages in OA, and the classical description of either pro-inflammatory (M1) or anti-inflammatory (M2) paradigm is not aligning with the functional status of tissue macrophages seen in OA. RNA-seq data from synovial OA shows a mixture of both M1- and M2-related genes can be expressed in synovial macrophages across OA (41). So, the traditional M1/M2 classification is no longer an accurate description for different types of synovial macrophages in OA and other inflammatory arthritis. Animal studies indicate that complex and multiple factors could be involved in the development of OA-like synovial insulin resistance (44), gut microbiota (45), and dietary fatty acid composition (46, 47). In a study to assess the synovial inflammation between early and late OA, the intensity of macrophage infiltration increased with increasing histological grade of OA (48). Multiple studies have implicated macrophage-mediated inflammatory response in the pathogenesis of OA (28, 49). However, Harasymowicz et al. (50) reported that the synovium and fat pad of obese patients had shown increased macrophage infiltration and higher toll-like receptor 4 (TLR4) gene expression. TLR4 recognizes LPS along with its extracellular components such as MD-2 and CD14 (51). Furthermore, there was an increase of CD14+ CD206+ M2- type macrophage in infrapatellar fat pad (IPFP) and ST. However, peroxisome proliferator-activated receptor γ and adiponectin were expressed in lower levels in IPFP and ST of obese patients compared to lean patients (50). These demonstrated that the M1/M2 paradigm does not fit with the obesity-induced macrophage activation. In aged mice, genetic deletion of TLR4 prevented the development of OA from high-fat diet-induced obesity (52). Multiple TLR single nucleotide polymorphisms (SNPs) have been implicated as genetic associations with knee OA like T-1486C SNP in TLR9 (53, 54), TLR3 SNPs (55), TLR7, and TLR 8 SNPs (56). These potential mechanisms need to be further explored to better understand the role of macrophages in obesity-related OA.

### 2.2 Neutrophils in Obesity and OA

In morbid obese individuals, it was demonstrated that the blood neutrophils have a low bactericidal capacity and adherence capacity (57). It is also demonstrated that neutrophil stimulation with LPS results in increased hydrogen peroxide (H_2_O_2_) production for pathogen clearance (58). Neutrophils are known to use high rates of aerobic glycolysis for ATP generation through hypoxia-inducible factor-1alpha (HIF-1a) (59). These activations of the HIF signaling pathway increase their survival, glycolytic metabolism, production of antimicrobial peptides, thereby effectively fighting against infection (58). Recent evidence demonstrates that in obese and asthma patients, obesity leads to shifting the Th2/eosinophilic response towards Th17/neutrophilic profile, contributing to the worsening of asthma (60). Xu et al. demonstrated the systemic effects of obesity in the human population by identifying the increased neutrophil percentage and increased expression of genes involved in neutrophil activation like neutrophil elastase and myeloperoxidase (57, 61).

Another study has shown that the release of basal superoxides, formyl- methionyl- leucyl- phenylalanine- stimulated superoxide, and opsonized zymosan-stimulated superoxides were elevated compared to that of lean controls (62). However, phagocytosis, CD11b surface expression, and adherence of neutrophils did not have any significant changes compared to the lean model. The study was drawn to a conclusion by observing the high level of superoxide production, chemotactic activity, normal phagocytotic activity, and adherence are significant signs of subjects with obesity carrying neutrophils being primed and having the capacity to fight infection. Since the neutrophils are in the prime state, they may participate in the pathogenesis of obesity-related diseases, including OA (62).

The role of neutrophils in OA pathogenesis is relatively unknown, whereas RA is well characterized. According to de Lange-Brokaar et al. (63), there are varying levels of neutrophils present in the synovium of OA. Hsueh et al. demonstrated the presence of neutrophils in synovial fluid through increased expression of elastase (43). Levels of elastase in synovial fluid are strong predictors of knee OA progression (43). Hsueh et al. found synovial fluid elastase and TGFβ are vital players in knee OA, reflecting the synchrony of neutrophils and macrophages population in the pathogenesis and worsening of OA (43). These studies do not have a consistent outcome on their results that could help in understanding the role of neutrophils in OA, further studies must be done to establish the independent role and their signalling cascade in obesity-linked OA.

### 2.3 Eosinophils in Obesity and OA

Eosinophils have a protective role in obese mice. Two prominent studies showed that an increase in the eosinophil numbers had reduced the fat in mice (64, 65). The increase in weight gain and glucose intolerance, this was associated with the reduced eosinophils (65). Contradictorily, in another study, the eosinophils population was increased in AT of high-fat diet-induced mice by chronic helminth infection and soluble helminth egg antigen. In treated mice, it was observed that the weight gain was less and less of fat mass gain, adipocyte size was smaller, glucose uptake improved, and AT insulin sensitivity also increased (66, 67).

Contradictory results were seen between mice and human studies with regards to the eosinophil activity in obesity. The positive coexistence of eosinophils with obesity was observed in an epidemiological study between body mass index or metabolic syndrome and blood eosinophil counts (68, 69). Similarly, Moussa et al. studies that the increase in the number of eosinophils in the SCAT was directly associated with metabolic syndrome (70). The study exhibited that circulating and SCAT eosinophils were two times higher in metabolic syndrome patients and correlated with each other. The findings were novel and seminal, where the eosinophils increased in SCAT in metabolic syndrome patients which is directly associated with the pro-inflammatory status. Therefore, it was concluded that dysregulation of SCAT biology contributes to metabolic syndrome in humans (70). A specific population of eosinophils was identified in patients with RA however a proper understanding of eosinophils and their functions in OA is still under investigation (71). A detailed study is needed to define how this innate immune cell type reacts when the joint microenvironment homeostasis is affected by this multifactorial disease. The role of eosinophils in obesity and OA has been elucidated in **Table 1**.

**Table 1:** Details cellular functions of the innate and adaptive immune cells on the review are mentioned in detail along with the references.

IMMUNE CELL AND ITS CHARACTERISTICS	OBESITY	OA
**INNATE IMMUNE SYSTEM**		
Increased ATM population and pro-inflammatory state	(26)	
Role of LPS and TLR 4 in macrophage activation and pro-inflammatory state		(50, 52)
Role of PPAR y in macrophage activation	(37, 38)	(50)
NK cell dysregulation	(72, 73)	(74)
Dendritic cells activation	(75)	(76)
Heterogeneity/Phenotypic switch M1/M2	(26, 30, 33)	(41, 50)
**ADAPTIVE IMMUNE SYSTEM**		
Th9 cells and IL-9		(77)
TH2/Eosinophil to Th17/Neutrophil transition	(60)	
Increased Th1 and IFN -y production	(78, 79)	
Role of PPAR-Y in T cells	(80)	
Biphasic expression of Th17 in obesity	(81, 82)	
Role of IL-17/Th17 in arthritis		(83–86)
Negative regulation of adipogenesis by IL-17		
Inhibitory and anti-inflammatory role of Treg in obesity and metabolic syndrome	(79, 87)	
Lack of Tfh in OA		(88)
Regulation of Th17/Treg in obesity/hypoxia	(89–91)	
Treg influence of M1 infiltration in AT	(92)	
Cytotoxic T cells	(93–95)	(96)
Role of CD8+ T cells in macrophage recruitment in obesity	(97)	
B cell – autoantibodies production	(98)	(99)
B cells infiltration	(100)	(99, 101)

### 2.4 DCs in Obesity and OA

DCS are specialized antigen-presenting cells that link adaptive and innate immune responses. DCs are a heterogeneous collection of cells distinguished by differential expression of essential transcription factors such as IRF8 and IRF4. This system recognizes two types of conventional/myeloid DCs (cDCs) and the plasmacytoid DCs (pDCs) (102). Myeloid cDC1 promotes the T helper type1(Th1) and NK responses through IL-12 and activates CD8+ T cells through MHC class I (103). Myeloid Cdc2 in human blood responds well to LPS, flagellin, Poly IC and R 848 as they are well equipped with a wide range of lectins, TLRs, NOD-like receptors, and RIG-I like receptors (104). Human Cdc2 is stimulated and produces huge quantities of IL-12 compared to Cdc1. Cdc2 has been shown as potent activators of Th1, Th2, Th17, and CD8+ T cells. The third type of DCs called the inflammatory dendritic cells, defined as monocyte derived DCs, are seen in the inflammatory state. In humans, these cells are demonstrated in various settings like synovitis, psoriasis, inflammatory bowel disease (103). The heterogeneity of these cell surface markers makes it difficult to develop a clear consensus on other immune cells like monocyte, macrophage, or DCs to describe these cells. Segura et al. demonstrated that inflammatory DCs induce Th17 cell differentiation through stimulation of memory CD4+ T cells to produce IL-17 (105). They further proposed that these cells are derived from monocytes and are involved in the induction and maintenance of Th17 cell responses (105). Activated DCs were demonstrated to be increased in numbers in AT of obese non-diabetic humans compared to lean subjects (75). These activated DCs regulated the AT inflammation by regulating the switch towards Th17 cell is insulin resistance associated with obesity (75).

TLR family plays a fundamental role in the activation of DCs in OA, especially TLR4, promotes obesity-induced OA in the mouse model (Tab.1) (52). In the experimental OA model, increased DCs were found to significantly upregulate TLR 1-8 mRNA levels (76). TLR3 was significantly elevated in DCs from these experimental OA models in mice. These data suggested that the inflammatory activity of DCs in OA occurs through activation of membrane TLRs.

## 3 Adaptive Immune Cells in Obesity and OA

Lymphocytes constituted 10% of non-adipocyte cells, including T cells, B cells, NK cells, NK T cells, and ILC2. In mice models, deficient mature lymphocytes revealed greater weight gain than WT mice fed a high-fat diet (HFD). DIO mice lacking ab T and B cells led to worsening the VAT and skeletal muscle inflammation compared to WT DIO mice (106). During the development of obesity, there is a relative increase of CD8^+^ T cells and a decrease in Treg cells. Both CD4^+^ and CD8^+^T cells play a crucial role in the recruitment of ATM and polarisation (97). The accumulation of CD8^+^ T cells into AT precedes macrophage infiltration (79). Duffaut et al. demonstrated that human AT lymphocytes and murine AT lymphocytes differ in the relative proportion of T lymphocyte subsets (107). Compared to murine AT infiltration, there is no adipocyte production of CXCL12 in human subcutaneous white AT (SWAT) or VAT (107). However, in Class II/III obese subjects instead of CXCL12, there is increased expression of CCL20, CCL20 receptor (CCR6) in VAT (108). Various adaptive cells roles are discussed elaborately to understand how they are differentially expressed from the normal to in obesity and OA microenvironment. The **Figure 1** shows the adaptive immune cells and their upregulation/downregulation in physiological inflammation and pathological inflammation.

### 3.1 NK Cells in Obesity and OA

Several studies have shown inconsistent outcomes in terms of increasing or decreasing NK cell numbers. These discrepancies could be correlated with strain/species-dependent of the rodent models and their metabolic attributes or differences in the development, degradation, or migration of the NK cells (109–111). Compartmental NK cell distribution has also been studied along with the discrepancies mentioned above. The study found an increased number of NK cells in the blood and spleen but a decreased amount in the liver tissue of obese rats compared to lean littermates (43). A human study on the high BMI(>40kg/m2) obesity group demonstrated that both NK cell levels and functions were significantly compromised compared to lean subjects (72). The NK cells in obesity are characterized by decreased production of IFN-γ, granzymes, perforin, and reduced numbers (73). However, these characteristics are reversed by weight loss surgery and exercises (73).

Huss et al. demonstrated that NK cells infiltrate the synovium, but they are characterized by a quiescent phenotype which is consistent with post-activation exhaustion (74). These cells are found to have lost the capability to produce IFN- γ on cytokine stimulation (74). Recently, Jaime et al. has demonstrated the characteristics mentioned above of limited cytotoxicity of NK cells in the synovium of OA compared to peripheral blood (112). These NK cells in synovium expressed a lower amount of granzyme B and perforin (112). ATM express the NK group 2D (NKG2D) ligand Rae-1, making them a target for NK cell lysis (73). Babic et al. demonstrated that NKG2D promotes increased Th1 and Th17 pro-inflammatory production and causes antigen-induced arthritis (113).

NK cells secrete antimicrobial peptides like LL-37 (114). These are critical antimicrobial agents expressed on the surface of epithelial cells, which act as a barrier against bacterial invasion. The functional characteristics of these antimicrobial peptides produced by NK cells need detailed study with regards to obesity and OA.

### 3.2 T Cells in Obesity and OA

Obesity is promoted by T cells by recruiting macrophages into AT (97). In one of the studies where they stimulated T cells from an obese individual, it was found the T cells had limited insulin binding in comparison with lean individuals. Furthermore, the T cells obtained from obese individuals with type 2 diabetes mellitus (T2DM), expressed 40% fewer insulin receptors in comparison to obese individuals without T2DM. It was inferred that T2DM exacerbates defects due to obesity (115, 116).

Infiltration of T cells in the joints is the hallmark of OA. At any given time, healthy joints have few tissue-resident T cells within the synovium or in synovial fluid (117). Furthermore, the joint homeostasis is maintained by these tissue-specific T cells, infiltration of the pathogenic T cells occurs only when there is an inflammatory event. This activation of these cells could be in both antigen-dependent and independent cascading. The presence of both mono and oligoclonal T cells points towards antigen-specific proliferation (7). Moreover, the proliferation of T cells in response to the chondrocytes and synoviocytes membrane antigens also was existing in the circulation of some OA patients (118). Certain amino acid sequence from aggrecans is recognized in a few OA patient T cell population, these aggrecans constitutes a major part of the normal cartilage, but also be prone to autoantigen production within the joints (119). All the above data suggest OA is driven by the joint-derived antigen that is followed up by the production of the aberrant systemic and local T cell population. Haynes et al. in 2002 demonstrated that the presence of large cellular aggregates in OA synovial membrane in expressed T cell markers associated with immune activation and antigen presentation (120). T cells are predominantly found in the sub-lining layer of the synovium and to some extent in the deep layer (19, 40). The MNC infiltrates found in OA consist of T cells expressing early, intermediate, and late activation antigens (121). However, the decreased expression of CD3 zeta protein in OA suggests chronic T cell stimulation (122). It is found that CD80, which is an inducible co-stimulatory ligand involved in T cell stimulation, is expressed in synovial aggregates from OA (120). A relative abundance of CD4^+^ T cells is found in OA ST with a CD4^+^/CD8^+^ T cell ratio of 5:1 compared to 2:1 in normal synovium (8). This shows that the T helper cells are potentially involved in the pathogenesis of OA. Different types of T cells and their role in obesity and OA are summarized on **Table 1**.

#### 3.2.1 Th1/Th2 Cells in Obesity and OA

IL-12 stimulates the naïve T cells to differentiate into Th1 cells. In VAT and SAT obese and non- T2DM human subjects have a 10-20 fold greater frequency of Th1 than Th2 (123). Increased frequency of Th1 in VAT and SAT is correlated with IL-6 and CRP levels (27). Under hypoxic conditions, Th1 cells lose the capability to produce IFN-γ through a HIF - dependent manner (124). The low oxygen tension state will activate HIF-1, which leads to phosphorylation of STAT3, a transcription factor for IL-17 and differentiation to Th17, and inhibition of Th1 (124, 125). Khan et al. demonstrated that T cell deficiency is associated with reduced IFN-γ levels, reducing AT inflammation and metabolic dysfunction (106). Higher levels of IFN-γ promote Th1 polarisation, this is associated with waist circumference (126).

In OA, there is no variation in cell count numbers of circulating Th1 cells in the peripheral blood compared to healthy controls. However, there is an increase in Th1 cells in OA patients in the synovial fluid and synovial membrane. These cells are found in the sub-lining layer of synovium and IFN-γ^+^ in nature compared to IL-4^+^ cells (127). The origin of IFN-γ needs to be explored further in OA to identify the cell of origin. On the contrary, in OA condition, current studies reveal that Th2 cells play a limited role in pathogenesis (127). This is demonstrated through multiple studies showing minimal alterations in Th2 cells in peripheral blood, synovial fluid, and synovial membrane. There is also evidence of low concentrations of IL-4, IL-10 levels in synovial fluid, and there is an absence of IL-4 and IL-5 in the synovial membrane of OA patients (121). A clear subpopulation study will help in establishing whether Th1/2 cells could aid in managing OA.

#### 3.2.2 Th17 in Obesity and OA

Th17 is a subset of CD4^+^ T cells characterized by the secretion of pro-inflammatory cytokines like IL-17, IL-22, and IL-21 and plays an essential role in various autoimmune diseases (128). Th17 cell differentiation is induced by TGF- β, IL-6, IL-21 and maintained by IL-23 (81, 128). Ivanov et al. (129) demonstrated retinoic-acid receptor-related orphan γt (RORγt), an orphan nuclear receptor as the key transcription factor in the differentiation of Th17 cell lineage. RORγt induces genes encoding IL-17 and thus the manifest response to IL-6 and TGF-β. Bettelli et al. (Tab.1) (89) delineated that IL-6 can completely inhibit the generation of T reg cells induced by TGF- β and IL-23 is independent of the above cytokines in the polarisation of naïve CD4^+^ T cells (89). However, Yang et al. demonstrated that STAT3 activated by both IL-6 and IL-23 played a critical role in the development of Th17 cells (125). Moreover, STAT3 regulated the expression of RORγt, and deficiency of STAT3 resulted in impaired RORγt expression, which led to elevated expression of T-box (Th1) and Forkhead box P3 (T reg cells) (125). Thus TGF-β has a dual role in inducing the anti-inflammatory T regs or the pro-inflammatory Th17 cells depending on the IL-6 state (89, 129).

Winer et al. demonstrated the increased presence of Th17 cells in diet-induced obesity and then on numerous studies confirmed the increased Th17 bias (128) (82). Interestingly, IL-17 shows a biphasic response in obesity and T2 DM development where at later stages IL-17 levels decrease, thus predisposing to increased adipogenesis (128). Transcriptional profiling of Th17 cells revealed increased acetyl CoA carboxylase 1 (ACC1) is an essential regulator of Th17 differentiation *in vitro* and pathogenicity *in vivo* through modulating RORγt (83). Thus, ACC1 forms the link between fatty acid synthesis and Th17 obesity-related pathology regulation. IL-17 functions as a negative regulator of adipogenesis and glucose metabolism and helps in delaying the development of obesity (90).

However, in hypoxia conditions, increased expression of HIF-1a promotes Th17 cells production and reduces T reg cell expression (Tab.1) (91). When T cells are activated through antigen stimulation, there is a metabolic switch to glycolysis mediated through HIF-1 in Th17 cells and not in T reg cells (91). Furthermore, Th1 and Th2 cell differentiation were largely independent of HIF1a, but HIF1a deficiency significantly impaired Th17 differentiation and IL-17 production (91).

Garidou et al. demonstrated that IL-17/RORγt deficient CD 4^+^T cells could induce T2DM and obesity (130). A HFD induces ileum dysbiosis and reduces antigen-presenting ability to induce Th17 cell differentiation. However, HFD feeding can stimulate Th17 cell development in the spleen, thus accelerating the onset of some autoimmune diseases like collagen-induced arthritis (83, 84).

In OA, it is widely accepted that Th17 cells are present in synovial fluid and ST (127). However, there is a discrepancy in the circulating Th17 cells in OA vs healthy group. These discrepancies require further investigation and potentially could explain the biphasic role of IL-17 in obesity. IL-17 can induce the production of IL-6 and IL-8 (ligand for CXCR2) through its effects on synoviocytes or normal skin fibroblasts, leading to recruiting cells of granulocytic lineage like neutrophils and protecting against bacterial infection (131, 132).

There are two contradicting studies regarding the expression of Th17 cells, in OA microenvironment, Zhang et al. demonstrated no difference between circulating Th17 cells or IL-17 plasma levels between OA and healthy controls (133). However, recently Qi et al. demonstrated in a study of 25 OA patients that the number of circulating Th17 cells and IL-17 levels are significantly elevated in the OA group compared to healthy controls (85). A study comparing RA and OA ST expression of Th17 cells found that the frequency of Th17 cells is elevated in OA but lower than RA (Tab.1) (86). More studies are required to have a better understanding of the role of Th17 and IL-17 in OA.

#### 3.2.3 Cytotoxic T Cells in Obesity and OA

CD8^+^ T cells are less prominent in VAT in humans compared to CD4^+^ T cells, but CD8^+^ T cell numbers in VAT are positively correlated with the BMI of subjects. In obese mice models, The increased expression of HIF-1a is linked to an increase in CD8+ T cell influx and 2a and GLUT1 due to the hypoxic environment present in AT (93). mTORC1- HIF1 pathway controls the fate of CD8^+^ cytolytic T cells through glucose metabolism and glycolysis (94). This is a PI3K-Akt independent mechanism, and GLUT1 expression is linked to promoting the survival of macrophages by facilitating glycolysis in a hypoxic environment (93, 94). However, in mice models, CD8^+^ T cells exhibit perforin-dependent killing of dendritic cells and other T cells to limit abnormal T cell activation in a physiological situation (95, 134). This shows the biphasic characteristics of CD8^+^ T cells depending upon the environment.

In OA, even though helper T cells are abundant in ST, cytotoxic T cells occur sparsely in various layers of ST. In synovial lymphoid, aggregates found in OA CD8^+^ T cells are found in the periphery (120).In anterior cruciate ligament transection OA models, increased activation of CD8^+^ T cells are manifested, and these cells show increased expression of tissue inhibitor of MP1 (Tab.1) (96). Saejung et al. demonstrated that perforin production is significantly lower in the blood of OA patients compared to healthy subjects (135). Recently, follicular helper cell expressing CD8 instead of CD4 has been found in germinal centres of SLO (136), so a more in-depth analysis of CD8^+^ T cells needs to be done to differentiate the type of cells activated in OA.

#### 3.2.4 Treg Cells in Obesity and OA

T reg cells are found highly enriched in visceral AT of lean mice, but it is markedly reduced in obesity and insulin resistance (Tab.1) (87). iNKT cells in VAT regulate Treg cell homeostasis, and innate lymphoid cell group 2 (group 2 innate lymphoid cells ILC2s are a recently discovered subpopulation of innate lymphocytes with critical immunological and homeostatic roles in many organ locations, particularly the lung. These cells are found in the lung and other peripheral organs, and they grow locally after birth and during postnatal lung development) controls Treg cells through a direct interaction of co-stimulatory molecules such as (ICOS) and ICOS ligand (137, 138). Under the influence of TGF-β, naïve T cells differentiate into Treg cells (138, 139). Treg produces IL-10 and thereby suppresses the inflammation and maintains insulin sensitivity. IL-10 limits the M1 macrophage infiltration of WAT by suppressing monocyte chemotactic protein -1 (MCP-1) (Tab.1) (92).

In non-obese OA, Treg cells were found in increased numbers in peripheral blood, but on the other side, lower secretions of IL-10 were noticed from Treg cells (140). This drop-in IL-10 is linked to lower levels of T cell immunoglobulin and mucin domain-containing protein 3, a checkpoint receptor similar to PD-1 (140). Moradi et al. demonstrated increased T reg cells in both OA and RA ST and activated effector memory phenotype (141).

#### 3.2.5 Th9 in OA

IL-9 producing Th cell subsets have been identified recently, and they are closely associated with autoimmune responses in RA, EAE, and systemic lupus erythematosus (142). These cells and a higher level of IL-9 have been detected in the synovial fluid and peripheral blood of RA and psoriatic arthritis (PsA) patients (77). In the same study, IL-9 levels are increased in peripheral blood and synovial fluid in patients with OA but not as high as RA or PsA patients (77). Roy et al. recently demonstrated that Th9 cells are highly glycolytic compared to other Th cells. Th9 cells differentiation is further enhanced under hypoxic conditions (143). Also, Th9 cells generate a significantly large amount of ATP through the glycolytic pathway, and this increase in glycolysis is brought in by the mTOR-HIF-1a signaling pathway (143). There are no studies conducted in obese tissue samples for Th9 cell and IL-9 expression. Investigation into the tissue specificity of Th9 cell lines in obese conditions could unravel essential answers in establishing a bridge between obesity and OA.

#### 3.2.6 T Follicular Helper Cells (TFH) in OA

TFH cells are found in lymphoid tissue follicles, where they stimulate B cells to generate immunoglobulins. CXCR5, PD-1, ICOS, CD40L, Bcl-6, and IL-21 are among the genes expressed by these cells (130). Shan et al. recently discovered a greater number of TFH cells in the peripheral blood of OA patients compared to healthy people (Tab.1) (84). They discovered that in OA, greater levels of serum IL-21 and expression of IL-21^+^TFH cells were linked to disease activity (84). Zhu et al. found an increased number of TFH cells in OA, which were positively linked with CD3^+^CD4^+^CXCR5-PD-1^+^ T cells and Th17 cells in a comparable investigation utilizing peripheral blood (131). However, in a study of ST by Chu et al., there are no signs for TFH cells in both OA and normal tissue ST samples compared to RA (Tab.1) (88).

In the hypoxic condition, TFH cells are regulated by mTOR complexes 1 and 2 through HIF. Cho et al. demonstrated the role of HIF in the interaction between CD4+ T cells and germinal centre (GC) B cells (144). In TFH cells, HiF2a induces the expression of CD154, which is essential for the stimulation of CD40 on GC B cells (144). In obesity models, there is a differential expression of TFH in secondary lymphoid organs and other tissue regions (60). A detailed study in the tissues obtained from overweight/obese OA patients would throw light on uncovering the signalling cascading involved in the development of OA in obese patients.

### 3.3 B Cells in Obesity and OA

Different B-cell subpopulations in humans can be identified in peripheral blood and other organs by differential expression of various surface markers. These various subgroups represent various levels of development, activation, and differentiation. The B cell regulation of T cells occurs through the secretion of pro-inflammatory cytokines and pathogenic autoantibodies (IgG class) (145). In mice models, B1 cells are shown to produce IgM (natural antibody), which are anti-inflammatory, whereas B2 cells seen in VAT produce IgG that are pathogenic cells associated with obesity-associated inflammation (100). B1 cell-associated IgM production in AT is inversely correlated with circulating monocyte chemoattractant protein1 such that it blunts M1-like macrophage-mediated inflammation in DIO mice models (100). Furthermore, B1 derived IgM antibodies exhibit both direct and indirect anti-inflammatory activities and are considered to protect against diet-induced chronic inflammation in some cases (146–148). As B1 cells are very rich in omental AT, it is an essential regulator for VAT function (149). However, unlike B-2 cell–derived IgG, which exacerbates inflammation, B-1 cell–derived natural IgM inhibits inflammation (150–153). B-1 cells have been shown to reduce VAT inflammation, glucose intolerance, and IR in diet-induced obese mice (100, 154). A detailed study into the role of B cells subpopulation is required to understand their role in obesity which ultimately will aid in understanding their role in OA.

In classical OA, B cells are found in low numbers in ST compared to RA (63). However, it is reported that OA ST has relatively more B cells inflammatory infiltrates. These infiltrated B cells in ST were oligoclonal, suggesting an antigen-driven expansion (155). Furthermore, sequencing of complementarity determining regions of B cells indicated that these cells had been clonally expanded (101). In a high-fat obese mouse model, Schott et al. demonstrated early synovial B cell infiltration and activation of numerous inflammatory pathways. These B cells are potentially considered novel mediators of early obesity-associated OA (Tab.1) (99). The activated B cells will become plasma cells, leading to increased antibody production (101). Multiple studies revealed auto-antibodies against cartilage-derived proteins in OA like osteopontin, cartilage intermediate layer protein (CLIP), YKL-39, fibulin, and collagen (156). Antibody production plays a major role in any disease, epically OA as inflammation is a huge influencing factor. This statement comes as contradictory evidence to the role of regulatory B cells (Breg) which are proven to suppress inflammation in various diseases, including RA (157). Th study found that in SF, the B cells producing IL10 were directly present in the ex vivo and increased upon stimulation, this proves that one of the main sources of IL10 is B cells and affects OA patients. Furthermore, the study also analysed the functional analysis of blood to investigate IgM^+^ CD27^+^ B cells in OA patients. These IgM^+^CD27^+^ B cells were observed to secrete increased levels of IL10 but decline in the levels of CD80 and CD86 in comparison with non- IgM^+^CD27^+^ B cells. The blood IgM^+^CD27^+^ B cells were found to suppress the production of IFN γ which is an autologous expression of T cells, this could be a reaction to an over production of IL10. Moreover, they found that OA patients had lower levels of IL10^+^ B cells in the synovial fluid. When the study was concluded that IgM^+^CD27^+^ B cells subset in OA patients denoted as the major source of IL10 secreting B cells type in the SF and had the capacity to regulate functions in OA (157). So, a very close study on signalling cues will establish a clear understanding the role of B cells in obesity and OA, which could also eventually lead to developing intervention in the early stages of development in OA.

## 4 Other Influencing Factors

### 4.1 Mechanical Loading and Inflammation

Obesity induces a number of pathological changes to the whole knee joint structure, including abnormal loading on the joint, joint malalignment and muscle weakness (158). Obesity has long been associated with unequal distribution of the mechanical loading in the knee joint apart from the disruption in the physiological condition caused due to inflammation (158). Although “inflammation is a helpful process meant to confine and destroy threats to the host organism,” prolonged inflammation-induced changes in joint homeostasis in obesity-induced OA may decrease inflammation resolution and contribute to tissue regeneration failure (159). The articular cartilage is one such essential structure in the joints that lacks regenerative capacity when under abnormal acute or long-term mechanical loading. Under these abnormal conditions, they are prone to lesions that lead to OA (160). Various studies have shown that mechanical loading has led to the activation of inflammatory pathways and their channels like IL-1β, TNF-α, NF-κB, Wnt, microRNA, and oxidative stress pathways (160, 161). These are the few of the main pathways that lead to regulating joint inflammation, activating key degradation enzymes in articular cartilages such as MMPs and aggrecanases, including chondrocyte apoptosis, extracellular matrix (ECM) degradation, subchondral bone dysfunction, and synovial inflammation which ultimately lead to OA (161). Chondrocytes maintain the catabolic and anabolic process homeostasis, slowly turning over the cartilage extracellular matrix. Progressive cartilage degradation indicates the chondrocyte imbalance, which ultimately favors the catabolic processes. The activities of chondrocytes are influenced by soluble mediators, like growth factor and cytokines, local matrix composition, and biophysical factors, which also includes mechanicals (sensed by mechanoreceptors) or osmotic stress (162). Wang et al. compared the preserved with damaged cartilage, they identified the levels of estrogen receptor- α (ER- α) was significantly low in the damaged cartilage in comparison with preserved cartilage in both human and mice samples (163). Furthermore, they used a 3-dimensional culture model, when the induced mechanical loading suppressed the level of ER- α in the chondrocytes that lead concomitant upregulation of OA phenotype. The study demonstrated the independent role of ER- α with respect to mechanical loading and its effect on the chondrocyte phenotype (163). Clinical and animal studies revealed that increased joint loading, whether acute or cumulative contact stress, might affect the composition, structure, metabolism, and mechanical characteristics of articular cartilage, subchondral bone, and other joint tissues, ultimately leading to OA (78, 162, 163). Moreover, there was a clinical study conducted using 3D giant analysis, where the 32 young obese individuals and 16 normal weight age-matched individuals were tested based on the mechanics of knee and ankle joints. The analysis was based on kinematic and kinetic data which revealed that knee flexion was less, greater knee ab- adduction angle during the gait cycle test, and knee flex-extension moment abnormalities were observed. Reduction in the range of motions together with a lower peak of ankle plantarflex or moment and power during terminal stances at the ankle joints were noted (164).

### 4.2 Role of AT Metabolism in Obesity and OA

The organization of white AT is found in various depots in the body, this includes under the skin (subcutaneous), within the abdominal cavity (visceral) and in small depots within the most organs. Statistics show that up to 10- 20% of the adipose in men are depots in the visceral and 5-8% in women (165). Moreover, the inclination of developing type 2 diabetes and metabolic syndrome is strongly associated with the accumulation of visceral fat (166–168). In obesity-associated metabolic syndrome, the AT lipid storage dysfunction leads to rising in the level of circulatory free fatty acids which gets reposited in visceral fat, liver, muscle, and pancreatic β- cells which leads to the deposition of ectopic lipids that causes lipotoxicity (169). The highlighted feature of lipotoxicity is an accumulation of reactive lipid aldehydes. This includes the 4- hydroxynonenal and malondialdehyde and subsequent protein carbonylation (170). In addition to lipotoxicity, changes in antioxidant enzymes activity, expression, and particular gene variations, as well as oxidative alterations of mitochondrial DNA (mtDNA), have been examined as possible indicators of metabolic disorders. Reduced activity of antioxidant enzymes, including superoxide dismutase (SOD), catalase (CAT), and glutathione peroxidase (GSH-Px), as well as a substantial drop in the GSH/GSSG ratio, were detected in obese individual peripheral blood mononuclear samples (171).

Previous studies have shown that more inflammatory cytokines are secreted into the circulation with excess fat secretion, with higher levels of adipokines and inflammatory protein in obese individuals. Furthermore, the cellular interaction between macrophages and adipocytes resulted in adipose-associated inflammatory responses. The characteristic M1 phenotype for obesity has been known to increase the production of adipokines, and in turn adiponectin which regulates the obesity-induced inflammation in OA and modulates immune responses (12, 172, 173). The concentration of adiponectin was reported to be higher in concentration in OA patients in comparison with controls. Further studies have reinforced the concentration of adiponectin and leptin was closely linked to female gender, body mass index, and synovial inflammation, these findings pointed out the prospective role of adiponectin in the serious inflammatory components of OA (174). A study by Kroon et al. showed that levels of leptin in the serum has close relation with OA and is associated with the partial adiposity. However, adiponectin levels are not associated with OA condition (175). These findings have thrown a light on how AT has a major influence on the OA.

### 4.3 Role of Insulin Resistance in Obesity and OA

Obesity link to various diseases, particularly insulin resistance and type 2 diabetes mellitus (T2DM). Evidence based reporting suggests AT is very versatile in terms of their metabolic flexibility due to the energy demands and being able to cope with large and rapidly evolving balance between fasting and feeding through the day and also fine tune to long term changes in energy balance with tissue expansion and reduction (176). This versatility of the AT especially the feature of expansion and reduction is detrimental of the AT health and systemic metabolic homeostasis, and in changes to the responses are likely to a contributing factor to the heterogeneity in the metabolic health observed in people with obesity (177–179). The ground-breaking discovery in mice AT produces proinflammatory cytokines that causes insulin resistance and discovery of accumulation of AT macrophages in obese population, has led to hypothesis that adipose inflammation is a major driver of insulin resistance in obese population (25, 180). Even though, there are significant increase in inflammatory macrophages and gene expression of the proinflammatory proteins in subcutaneous abdominal AT in individuals with metabolically unhealthy obesity in comparison with metabolically healthy obesity, it is very hard to deter whether it is the cause or effect of insulin resistance (181). The free fatty acid concentration associated with obesity and T2DM could have an adverse effect on pancreatic β cells. 30% of insulin secretion is due to circulating free fatty acids in the basal condition in people with or without diabetes (182). Furthermore, there is a strong link that associates obesity and increase in the rate of free fatty acid in the bloodstream and delivery of it to the body tissues (183). Although, there are strong studies exhibiting the increase in the level of plasma free fatty acids concentration, these are important cause of liver and muscle insulin resistance, various evidence become questionable when conflicting data emerge with the real world scenarios in contrast to the experimental set ups (184). Several studies prove that lipolysis of AT triglycerides is very sensitive to insulin, the postprandial suppression of lipolysis and plasma FFA concentrations is often the same in lean and obese human subjects because the greater postprandial increase in plasma insulin in obese subjects can possibly make up for their increased fat mass (185–187). Insulin resistance and relation to obesity is still a conundrum which needs a clear pathway mapping relation between T2DM and increase in the inflammatory macrophages in subcutaneous layer AT.

The role of diabetes in OA has various contradicting view obtained from clinical data. A group from Puerto Rico et al. did a cross sectional study on 202 subjects, they found that patients with OA had diabetes mellitus were 49% however, only 26.5% of patients with diabetes mellitus had OA. The study also examined and proved that number of females with diabetes mellitus were more to have knee or hand OA were more than males. Various other aspects were also considered like the age, gender, education level, obesity, exercise, and osteoporosis, even with which the patients with diabetes mellitus had 2.18 times the risk of hand or knee OA in comparison with nondiabetic patients (188). A 3- years follow-up study was conducted to understand the gender effect of diabetes mellitus on OA. Out 559 patients who were over the age 50 or over the male patients with T2DM developed a narrower joint space than female (189).

On the contrary various other studies supported no link between OA and diabetes. A systemic review that consisted of 31 independent study were the sample space was 295,100 supported the notion of OA and diabetes are two independent factor and not only diabetes is a sole contributor to the development of OA, moreover, the study also suggested that the higher body mass is the main associate from inducing OA (190, 191).

The molecular signalling cascades between diabetes and OA has not been well explored. A study on extracellular glucose level on chondrocytes showed, low concentration of extracellular glucose concentration (5- 10mM) there was increase in the glucose transporter 1 (GLUT1) expression in healthy chondrocytes, when the concentration was increased (25- 75mM) the level of GLUT1 decreased significantly (192). Rosa et al. exhibited that chondrocytes isolated from health individuals were able to adjust to the fluctuation of high glucose levels. However, chondrocytes from OA patients were not able to regulate GLUT1 expression (193). Autophagy was reduced in diabetic group in both human chondrocytes and mouse studies which paves a new direction in understanding the cartilage degradation in diabetes condition with regards to defective autophagy. Rapamycin, a pharmacological activator of autophagy, reduced cartilage breakdown, proteoglycan loss, inflammation, and lowered MMP13 expression in experimentally produced db/db OA animals (194, 195). Both animal and human studies have yielded inconsistent data to arrive at conclusion whether diabetes is independent of OA or there could be unexplored pathways that should be examined for establishing a detailed pathways between diabetes and OA.

## 5 Anti-Inflammatory Therapeutics for OA

In this review there are extensive discussion on obesity and OA and the inflammatory immune profiling, however the influence of the drugs that aids in altering the immune profiling of OA is still under investigation. However, none of these interventions has been shown to significantly alter disease progression or successfully prevent eventual joint replacement in the advanced disease stages. Numerous drugs which influence the inflammatory pathways are being extensively studies, out of which few of their biologics and their alteration in OA is discussed below.

### 5.1 IL-1 Inhibitors

The expression of IL-1 has been extensively seen in cartilage, synovium, and SF in OA patients (196). The drugs that target IL family like the Anakinra and human IL-1 receptors type 1 monoclonal antibody AMG 108 are produced by genetic recombination (197, 198). A randomized trial with double blind, placebo control of AMG 108 was administered subcutaneously and intravenously every 4 weeks for 12 weeks. The patients showed insignificant improvement in OA symptoms but greater improvement in the pain control compared to placebo (197).

### 5.2 TNF-α Inhibitors

The proinflammatory cytokine, TNF-α produced by the synoviocytes and chondrocytes in OA, play a critical role in modulating the pain and structural damage in OA. Additionally, TFN-α is a key player in enhancing the production of proinflammatory cytokines like IL-6 and IL-8, also triggers the synthesis of MMP and cyclooxygenase and increases the level of NO production (199). Etanercept is one such drug that is a recombinant human necrosis factor type II antibody fusion protein. The study showed that the IA injection of etanercept compared with hydraulic acid, relived pain effectively in OA patients (200). However, A random double-blind placebo- controlled trail with subcutaneous injection of etanercept for 24 weeks did not show any pain reliving effects in patients with hand OA compared with placebo (201). Whereas etanercept treated for 52 weeks for joints showed radio graphical remodelling and less MRI bone marrow lesions (201). In this study the application of etanercept was observed to decrease the level of MMP3, an important mediator of joint destruction (202). Conclusion to the study was given by the author to use this drug for etanercept for short term as a treatment with TNF-α inhibitor during OA flare ups (203). There are other drugs that are considered for TNF-α inhibition, like infliximab and adalimumab. While infliximab does not have any clinical trials supporting possible symptom and disease alleviating effects (203). Adalimumab did not have any significant effect on the erosive hand OA, did not affect synovitis or the bone marrow lesions in hand OA with MRI detected synovitis (204, 205).

### 5.3 Resolvin

Resolvin D1 (RvD1) is derived from omega3 fatty acid which is known to be a specialized proresolving mediator which has been proven to have anti- inflammatory and antiapoptotic effects in OA (206). Furthermore, the study showed that the drug inhibited the production of OA- FLS by promoting the yes-associated protein phosphorylation and protects the chondrocytes *via* inhibition of IL1β and MMP13 production which provides a potential experimental treatment of OA (98). However, there are no *in vivo* studies that are currently available to prove their potential anti- inflammatory properties and apoptotic properties.

### 5.4 Mitogen-Activated Protein Kinases (MAPKs) Interference in Pro-Inflammatory Pathways

MAPKs has shown some significant results with regards to murine models and clinical trials. In a murine destabilising OA model, local administration of a strong p38 MAPK inhibitor (PH-797804) decreased joint degradation and inflammation (207). In a clinical investigation involving knee OA patients, the efficacy of PH-797804 was compared to naproxen, however the findings have not yet been released (NCT01102660). FX-005, another therapeutic p38 MAPK inhibitor with sustained-release kinetics, was tested in a phase I/II knee OA study and shown to be superior to placebo in terms of pain reduction after 4 weeks (NCT01291914) (208). Direct TLR targeting might give even more upstream interference with OA immune activation; for example, a miR-21 inhibitor targeting TLR7 was able to cause long-lasting analgesia in an OA rat model.

### 5.5 Macrophage Immunomodulation

Hyaluronic therapy was found to recruit more anti-fibrotic macrophages which also helps in decreasing the pain (209). In a similar manner, a recent study found that administering alpha defensin-1 makes macrophages anti-inflammatory to an extend and reduces OA in a surgical model (210). These findings show that targeted anti-inflammatory therapy soon following knee injury may represent a viable future therapeutic strategy, justifying further experimental and, eventually, clinical research in OA.

Macrophage immunomodulation of M1 to M2 has become more prominent anti-inflammatory therapy target for researchers. The study by Li et al. showed extracellular vesical derived from human umbilical cord could be a potential target for promoting the M2 macrophages and secretion of anti-inflammatory cytokine IL10 (24). Their animal studies showed that the miRNA mediated M2 polarization was taking place which indicated the immunomodulation potential (24). All these results indicate that immunomodulation of macrophages has high potential for translation into humans.

## 6 Summary

Obesity is a key risk factor for the development of OA, which disrupts immune homeostasis and causes joint inflammation. OA is a multifactorial disease where various factors like mechanical loading, inflammation, and repairing of the ongoing injury are orchestrated in synchrony. Multiple studies have shown that inflammation plays a critical role in the development and progression of OA. PAMPs or DAMPs may have a role in the immune system’s dysregulation in obesity, which can lead to OA or a higher risk of infection.

**Figure 2:**
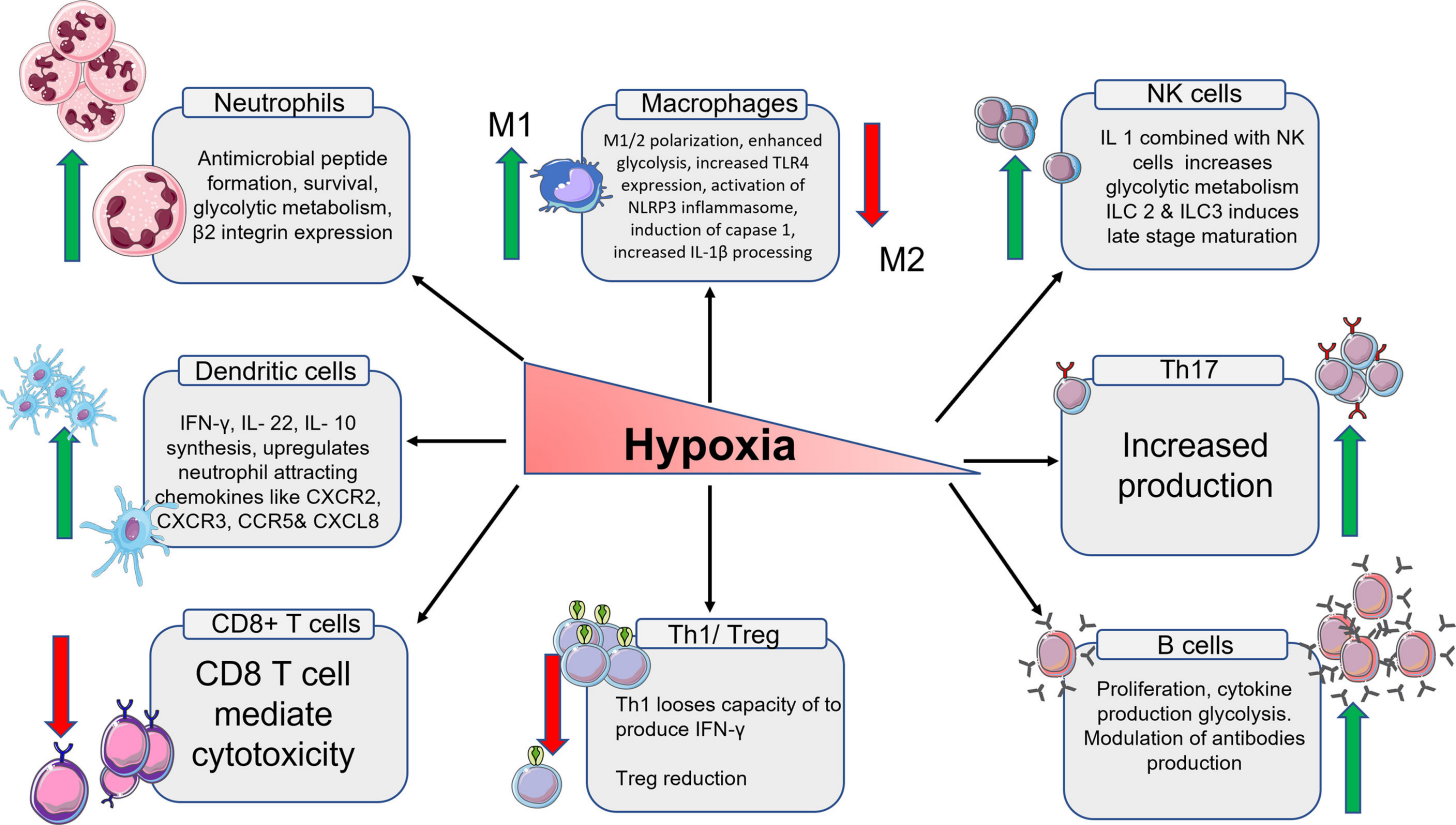
Schematic representation of the immune cells and their products in hypoxia condition. From left, the expression of neutrophiles cells multiplies. The expression of M1 macrophages increases and M2 macrophages decreases. The total number of NK cells goes up which in turn increases the glycolytic metabolism. The number of Th17 cells goes up. B cells also increases along with high regulation of antibodies. Th1 cells and T reg cells production decreases due to low oxygen. The number T cytotoxic cells also decrease because of reduced oxygen. The number of DCs increases which intern increases the production of chemokines associated with DCs.

The many phases of macrophage development and activation in terms of gene expression and chemokine secretion are discussed, as well as how these expressions differ from obese individuals in various animal models. There is a detailed look at the numerous signaling pathways that lead to M1 and M2 macrophage polarisation inactivation and insubordinate behaviour as a result of chronic inflammation brought on by obesity. There is little information about osteoarthritic pathology for myeloid origin cells such as neutrophils and eosinophils, there are few evidence-based research connecting to animal and human obesity. Especially the role of eosinophils is still unclear with OA pathology. There are no relevant studies that centres on the role of eosinophile in the ST during the development of OA. DCs are antigen-presenting cells that have been widely investigated for their role in the immune system as well as how they behave in obese mouse models. The subpopulation of DCs and their role in various cellular activation cues are well explained. It is also taken into consideration that these subpopulations might follow a similar path in OA as well. The role of all the innate and immune cells in hypoxia condition has been summarized on **Figure 2**.

All lymphoid cells that play a role in active immunity have detailed descriptions of their functions, activation, and signaling. Various studies on the involvement of NK cells in obesity physiology have produced conflicting results. Their significance in ST in the context of OA has a well-established signaling cascade. However, chemokines released by NK cells are said to have antimicrobial qualities, but there is not enough evidence to back up their claims or their role in OA physiology. Evidence for T cell subtypes is given in a complete and detailed study. Although the data for cellular signaling is widely given, it is insufficient to comprehend the multiple connecting signals that link obesity and OA, eventually leading to synovial inflammation.

According to the research presented in this review, B cells release IgG antibodies as well as cytokines to regulate T cells in obese models. Obesity-related chronic inflammation is exacerbated by these antigen-secreting cells. Nonetheless, a detailed literature review is necessary to fully comprehend their role in OA and obesity.

To comprehend the disease pathophysiology and determine the similar immunological pathways implicated in OA and obesity, more research on ST and the influence of obesity on OA in terms of immune cells and their activities is needed. In obesity, various pathways contribute to immunological homeostasis loss, such as intestinal dysbiosis or hypoxia, that need to be investigated OA. Furthermore, many other cytokines associated with OA have also become therapeutic targets. Most of the therapies target the pro-inflammatory pathways like IL-1β, TNF-α, resolving, MAPKs and macrophage immunomodulation therapies which could effectively reduce the inflammation in OA without side effects.

## Author Contributions

UN drafted, critically revised, and designed the figures for the manuscript. IV designed the outline and drafted the manuscript and the table. AS structured the abstract, reviewed the concepts and language usage. XW, IP, and RC all were involved in editing the document. All the authors contributed to the article and approved the submitted version.

## Conflict of Interest

The authors declare that the research was conducted in the absence of any commercial or financial relationships that could be construed as a potential conflict of interest.

The reviewer XL declared a shared parent affiliation with the author XW to the handling editor at the time of the review

## Publisher’s Note

All claims expressed in this article are solely those of the authors and do not necessarily represent those of their affiliated organizations, or those of the publisher, the editors and the reviewers. Any product that may be evaluated in this article, or claim that may be made by its manufacturer, is not guaranteed or endorsed by the publisher.

## Abbreviations

OA, Osteoarthritis; AT, Adipose Tissue; ST, Synovial Tissue; DCs, Dendritic cells; NK, Natural killer cells; RA, Rheumatoid Arthritis; ER- α, Estrogen receptor- α; FLS, Fibroblast-Like Synoviocyte; MMPs, Matrix Metalloproteinases; IPFP, Infrapatellar Fat Pad; SCAT, Subcutaneous AT; VAT, Visceral AT; SWAT, Subcutaneous White AT; PAMPs, Pathogen-Associated Molecular Patterns; DAMPs, Damage-Associated Molecular Patterns; PRR, Pattern Recognition Receptors; LPS, Lipopolysaccharide; IFN-γ, Interferon gamma; IL-#, Interleukins-#; iNOS, Inducible Nitric Oxide Synthase; WAT, White AT; TNF-α, Tumor necrosis factor-alpha; ATM, AT Macrophages; MMe, Metabolic Activation; TLR4, Toll-like receptor 4; SNPs, Single Nucleotide Polymorphisms; NKG2D, Natural Killer Group 2D; H_2_O_2,_ Hydrogen peroxide; HIF-1a, Hypoxia-inducible factor 1-alpha; RORγt, Retinoic-acid receptor-related orphan γt; ACC1, Acetyl CoA Carboxylase 1; T2DM, Type 2 Diabetes Mellitus; GC, Germinal Centre; RvD1, Resolvin D1; MAPKs, Mitogen-Activated Protein Kinases.
